# Selection of the Reference Gene for Expression Normalization in *Papaver somniferum* L. under Abiotic Stress and Hormone Treatment

**DOI:** 10.3390/genes11020124

**Published:** 2020-01-23

**Authors:** Zhaoping Zhang, Changjian Li, Junqing Zhang, Fang Chen, Yongfu Gong, Yanrong Li, Yujie Su, Yujie Wei, Yucheng Zhao

**Affiliations:** 1 China Agriculture Research System (CARS-21) and Gansu Provincial Key Laboratory of The Special Medine Source Plant for Germplasm Innovation and Safety Utilization, Gansu Academy of Agri-Engineering Technology, No. 234 Xinzhen Road, Huangyang town, Liangzhou District, Wuwei 733006, Gansu, China; gszzp4960@163.com (Z.Z.); ellychen123@163.com (F.C.); edwingyf@163.com (Y.G.); gsllyryr@163.com (Y.L.); syj2612179@163.com (Y.S.); gswwwyj67@163.com (Y.W.); 2 Department of Resources Science of Traditional Chinese Medicines and State Key Laboratory of Natural Medicines, School of Traditional Chinese Pharmacy, China Pharmaceutical University, Nanjing 210009, Jiangsu, China; lich12310@163.com (C.L.); junqingzhangcpu@163.com (J.Z.)

**Keywords:** *P. somniferum* L., reference gene, RT-qPCR, geNorm, NormFinder, RefFinder

## Abstract

*Papaver somniferum* L. is an important medical plant that produces analgesic drugs used for the pain caused by cancers and surgeries. Recent studies have focused on the expression genes involved in analgesic drugs biosynthesis, and the real-time quantitative polymerase chain reaction (RT-qPCR) technique is the main strategy. However, no reference genes have been reported for gene expression normalization in *P. somniferum*. Herein, nine reference genes (actin (*ACT*), glyceraldehyde-3-phosphate dehydrogenase (*GAPDH*), cyclophilin 2 (*CYP2*), elongation factor 1-alpha (*EF*-*1α*), glyceraldehyde-3-phosphate dehydrogenase 2, cytosolic (*GAPC2*), nuclear cap-binding protein subunit 2 (*NCBP2*), protein phosphatase 2A (*PP2A*), TIP41-like protein (*TIP41*), and tubulin beta chain (*TUB*)) of *P. somniferum* were selected and analyzed under five different treatments (cold, drought, salt, heavy metal, and hormone stress). Then, BestKeeper, NormFinder, geNorm, and RefFinder were employed to analyze their gene expression stability. The results reveal that *NCBP2* is the most stable reference gene under various experimental conditions. The work described here is the first report regarding on reference gene selection in *P. somniferum*, which could be used for the accurate normalization of the gene expression involved in analgesic drug biosynthesis.

## 1. Introduction

*Papaver somniferum* L., also known as the opium poppy, originated from Europe and now is grown around the world. It is famous as an analgesic, as well as a narcotic and alkaloid, and morphine, codeine, papaverine, and thebaine are its main compounds [1,2]. Due to their analgesic activities, pharmaceutical opiates are universally applied for relieving the pain caused by cancer, surgery, and wounds [3]. The opium poppy is also a source of narcotics, such as opium and heroin [4]. Consequently, many countries forbid or strictly control the cultivation of *P. somniferum* and the uses of opium products through legislation. However, *P. somniferum* is attractive for researchers, and research efforts have been undertaken in many aspects, including the pharmacological activity and metabolism of alkaloids in the plant [5,6].

For decades, plenty of studies have examined the biosynthetic pathway of *P. somniferum* [7,8,9,10]. With the development of sequencing technology, clarifying the biosynthetic pathway of this plant has become increasingly simple and convenient [11,12]. Additionally, another popular technology, RT-qPCR, is inclusively used for gene expression research due to its quantitative accuracy, high sensitivity, and high-throughput capabilities [13,14,15,16]. However, the results of RT-qPCR are unavoidably influenced by plenty of factors, including cDNA quality, RNA integrity, PCR efficiency, sample amount, and primer design [16,17,18]. To guarantee the credibility of RT-qPCR results, a stable and acceptable internal reference gene is required.

Housekeeping genes (HKGs) are frequently chosen as reference genes because of their predicted stable expression [19,20]. For example, *GAPDH*, *β*-*TUB*, *CYP2*, and 18s *rRNA* are often selected as reference genes. However, the expression levels of HKGs may vary between different species, and experimental conditions [21,22,23,24,25,26,27]. Thus, the selection of a stable reference gene has become increasingly important. In addition, according to previous studies, one or two extra reference genes are probably needed for normalization [14,16]. To evaluate the reference genes, algorithms and mathematical methods were developed to evaluate the stability of reference genes. The prevalent statistical processing tools, namely BestKeeper [28], geNorm [29], and NormFinder [30], are frequently used methods and were selected for this study.

In this study, nine HKGs from *P. somniferum*, *ACT*, *GAPDH*, Cyclophilin 2 *CYP2*, *EF*-*1α*, *GAPC2*, *NCBP2*, *PP2A*, *TIP41*, and *TUB*, were selected as candidate genes. Five different experimental treatments, cold stress (4 °C), drought stress (20% PEG), hormone stress (MeJA), salt stress (NaCl), and metal stress (CuSO_4_) were conducted. Finally, BestKeeper, geNorm, and NormFinder were used to analyze the gene expression stability. The results were further approved by the transcriptome data of *P. somniferum* according to its gene expression level. This study is the first systematic investigation of stable reference genes in *P. somniferum*, which could accelerate the research on *P. somniferum*, especially the gene expression involved in analgesic drug biosynthesis.

## 2. Materials and Methods

### 2.1. Plant Materials and Treatment

*P. somniferum* were collected from fields and transplanted into plastic basins which consisted of perlite, vermiculite, and peat moss at a ratio of 1:1:1. After planting for 5 months, the basins with plants were transferred into a greenhouse and grown for a week under suitable conditions, which is long photo period (16 h of light in 2500 lux and 8 h of dark) at room temperature (25 °C) with 40–60% relative humidity. For cold shock treatment, the plants were grown at 4 °C for 6, 12, and 24 h. For drought treatment, 200 mL of 20% PEG 6000 was subjected to the plants, and then the materials were collected at 6, 12, and 24 h successively. For salt treatment, 200 mL of 0.5 M sodium chloride (NaCl) solution was given, and collected the materials at 6, 12, and 24 h continuously. In hormone treatment, 200 mL of 25 mM methyl jasmonate (MeJA) was applied to treat the plants for 6, 12, and 24 h. Heavy metal treatment was conducted by CuSO_4_ solution, then collected the treated materials at 6, 12, and 24 h. In addition, one group of untreated plant was also harvested as control. After the materials were treated, the fresh parts of sample were washed and sterilized, and preserved in liquid nitrogen until use.

### 2.2. Selection of Candidate Reference Genes and Primer Design

Housekeeping genes are often opted as candidate reference genes because of their stability and housekeeping function in basic cellular processes [20]. In this study, protein sequences of nine candidate housekeeping genes (*ACT*, *CYP2*, *EF-1α*, *GAPC2*, *GAPDH*, *NCBP2*, *PP2A*, *TIP41*, *TUB*) from *Arabidopsis thaliana* L. were selected from TAIR database as templates (Table 1). Then, TBLASTN program in BioEdit was used to obtain nucleotide sequences of putative *P. somniferum* homologs from our transcriptome dataset [31]. The sequences used for primer design and their gene id can be found in Table S1. Then, the primers were designed by Primer 5 software according to following prescription: (1) melting temperature is about 60 ± 1 °C; (2) length of primers is between 20 and 30 bp; (3) GC content distribute from 40% to 60%; (4) amplification lengths from 100 to 150 bp. Primers used in this study are listed in Table 1. PCR efficiency was calculated by LinRegPCR and are also listed in Table 1. To guarantee the primer specificity, agarose gel electrophoresis of PCR products and melting curves was analyzed and the results are listed in Figures S1 and S2. In addition, the PCR products sequencing data of all the nine reference genes are also provided in Table S2.

### 2.3. Total RNA Extraction and RT-qPCR Analysis

Total RNA was extracted using EASY spin Universal Plant RNA Kit (Aidlab, Beijing, China), with which the gDNA can be eliminated thoroughly on the spin column. The reverse transcription reaction was processed using HiScript® II Reverse Transcriptase (Vazyme, Nanjing, China) in a 20 μL volume system containing 1 μg RNA. The reaction conditions were 50 °C for 15 min and 85 °C for 5 s according to the manufacturer’s protocol. The cDNA products were diluted five times with ddH_2_O and utilized as template for continuous qPCR reaction. The qPCR reaction system consisted of cDNA template, 0.2 μM of forward and reverse primers, 2 μL diluted cDNA, 10μL Hieff® qPCR SYBR Green Master Mix (YEASEN, Shanghai, China), and RNase-free ddH_2_O in a total volume of 20 μL. The reaction condition was set referring the recommendation from manufacturer, which is 95 °C for 5 min for 1 cycle, 40 cycles of 95 °C for 10 s, 55–60 °C for 20 s, and 72 °C for 20 s. The relative expression data was computed according to the 2^−ΔΔCt^ method [32]. 

### 2.4. Gene Expression Stability Analysis

To evaluate the stability of selected candidate reference genes, BestKeeper, NormFinder, and geNorm were employed. According to the manual, BestKeeper applies the raw Ct data and ranks the gene expression stability by calculating their coefficient of variance [28], the results were presented as CV ± SD. Different to the BestKeeper, data imported to NormFinder and geNorm must be converted to relative quantities by 2^−^^△Ct^ method [29,30]. GeNorm ranks genes by stability values by stability values (M value). Generally, genes with M values below 1.5 are acceptable for normalization. Besides, pairwise variation analysis (Vn/n+1) is also performed by geNorm for investigating the optimal number of reference genes for accurate normalization. The threshold is commonly set at 0.15 and additional reference genes are not required when Vn/n+1 is below 0.15 [29]. NormFinder employed a unique model for estimating intra- and intergroup variation [30]. For the three statistical methods, lower values always represent higher stabilities.

### 2.5. Statistical Analysis

To access the qPCR data, three biological and technique replicates were performed for each treatment. The raw qPCR data was analyzed by three mathematical methods. All graphs were generated by Origin 2019 (OriginLab Corporation, Northampton, MA, USA). Data were presented as mean ± S.E.M. (standard error of means).

## 3. Results

### 3.1. Expression Profile of the Reference Genes

**Figure 1:**
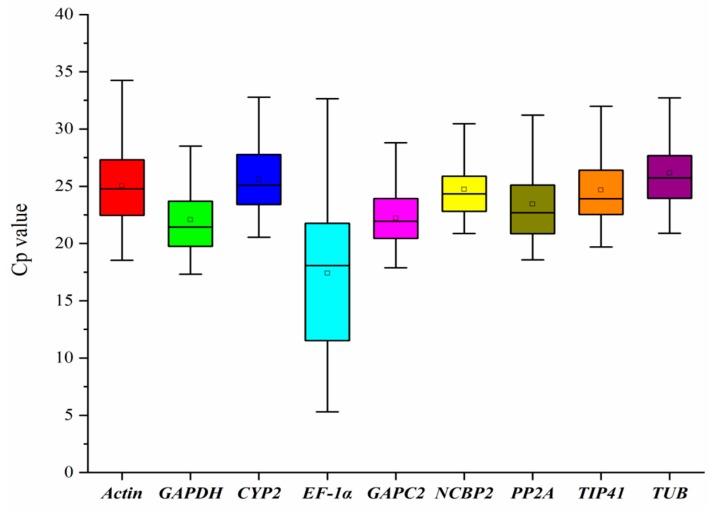
Distribution of cycle threshold values (Cp) of nine candidate reference genes. The expression data are demonstrated as Cp values for individual genes in samples of *P. somniferum.* The box graph shows the range of 25th to 75th percentile. The line across the box represents the median and the square in the box indicates the mean. The whiskers show the maximum and minimum values.

The cycle threshold values (Cp) represent the cycle number when the generated fluorescence signals reach a level that is able to be detected, and above 35 is considered to be undetectable. Hence, lower Cp values indicate higher expression levels; correspondently, a higher Cp value is equivalent to a lower gene expression level. As shown in Figure 1, the mean Cp values of the nine reference genes range from 17.60 to 25.87. The majority of the mean values were distributed between 20 and 27. *GAPC2*, *GAPDH*, and *EF-1α* have low mean Cp values while *CYP2*, *TUB*, and *ACT* have higher Cp values. Among them, *EF-1α* possesses the lowest mean Cp value of 17.60 ± 5.67, while *CYP2* has the highest mean Cp value with 25.87 ± 3.45. Gene expression variation is also shown in Figure 1. Obviously, *GAPC2* possess the lowest variability, with a narrow range of Cp values from 17.88 to 29.21, revealing that *GAPC2* might have a stable expression level under different treatments and could be chosen as one of the best reference genes. Conversely, *EF*-*1α* has Cp values that range from 5.3 to 32.65, so *EF*-*1α* is probably not a good choice for a reference gene. Through the Cp values, a boxplot not only determined the expression profile of the reference genes but also revealed their stability. However, considering the complexity of their surroundings, the stability of the reference genes under different conditions must be investigated thoroughly and systematically. Thus, more statistical tools and further analyses are required.

### 3.2. Expression Stability of the Candidate Reference Gene

In order to systematically evaluate the stability of the candidate reference genes, three commonly used Excel-based mathematics algorithms (BestKeeper, geNorm, and NormFinder) were chosen. The data were processed according to the requirements of the methods. In this way, a result with higher accuracy and credibility can be obtained.

#### 3.2.1. BestKeeper Analysis

BestKeeper is an Excel-Based algorithm that uses the raw data of RT-qPCR to evaluate the stability of candidate genes. In addition, BestKeeper can also validate the stability of a candidate gene by calculating its coefficient of variance (CV) and standard deviation (SD). Using this method, a lower CV ± SD value represents better stability, which means that the candidate genes listed on the top of the table are the most stable genes. As shown in Table 2, the outcome increases from the top to the bottom of the table. Accordingly, the *NCBP2* is the best reference gene under PEG and NaCl treatment with the lowest CV ± SD value of 7.66 ± 1.88 and 6.13 ± 1.45, respectively. In addition, *TUB*, *GAPC2*, and *TIP41* are considered good reference genes under cold, NaCl, and MeJA treatments. However, it is unacceptable to apply this as the final choice, as more analytical tools should be employed to obtain a reliable result.

#### 3.2.2. NormFinder Analysis

NormFinder is an algorithm used to select the most confident reference genes for individual experimental treatments. Unlike BestKeeper, RT-qPCR data, namely the Cp value, should not be directly applied to NormFinder. Instead, the raw data should be normalized by the ΔCt method, and then the data can be used for a NormFinder analysis. Similar to BestKeeper, a gene with a lower value indicates better stability. As Table 3 shows, from the top to the bottom of the table, the stability values of the candidate genes are ranked from lowest to highest, and the results under different experimental treatments are also listed. We can see that *PP2A* is the most stable gene under cold treatments, while PEG has the lowest values of 0.627 and 0.136. *TIP41* ranks in the top three under all treatments except CuSO_4_ and WT. On the other hand, the *EF-1α* is the most unstable candidate gene under all treatments with a stability value above 3. Moreover, under all treatments, *NCBP2*, *GAPC2*, *TIP41*, and *PP2A* can be recognized as the most stable candidate genes, which can be considered the best reference genes by NormFinder. Among all candidate genes, *NCBP2* can be instantly considered as the best reference gene by NormFinder analysis. This outcome further indicates that *NCBP2* is likely the most stable reference gene.

#### 3.2.3. geNorm Analysis

**Figure 2:**
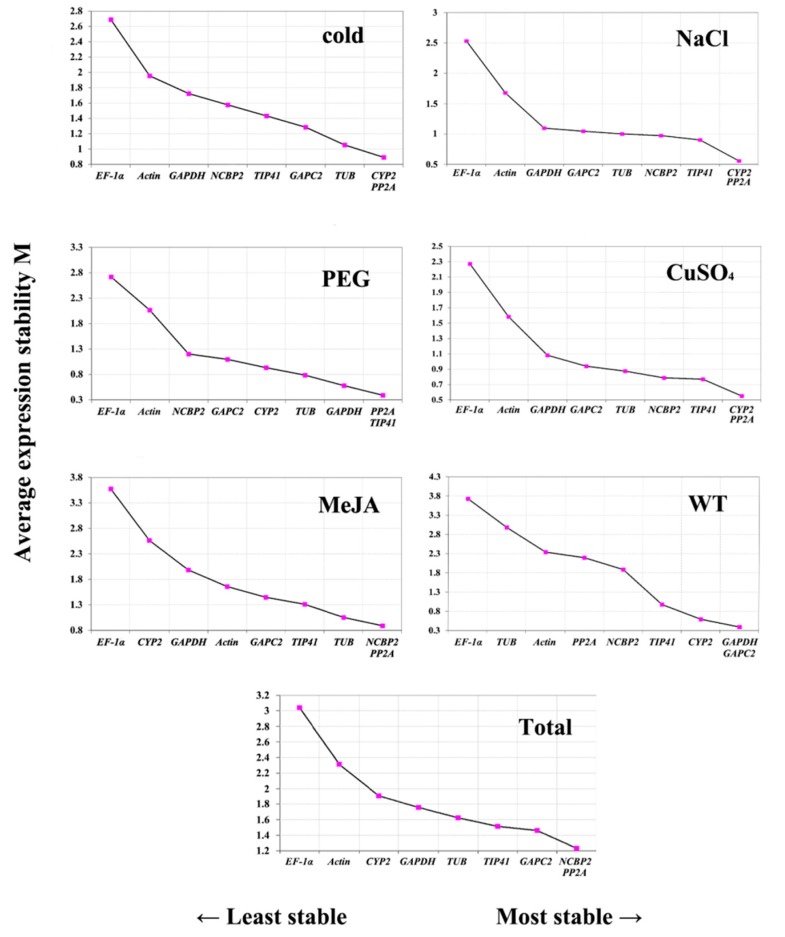
Average expression stability (*M* value) of nine candidate reference genes by geNorm. The candidate genes are listed from left to right. M values are listed from the top to the bottom. Treatments are noted on the top right corner of each group.

**Figure 3:**
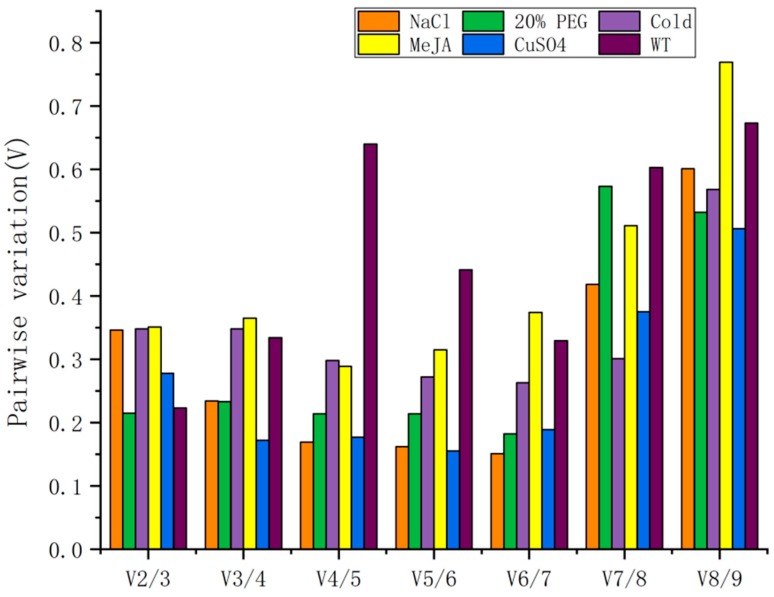
Pairwise variation result given by geNorm. The *V*-values were calculated by normalization factors NF_n_ and NF_n+1_, suggesting the optimized number of required reference genes for normalization.

geNorm is another good tool for expression level research. geNorm ranks the expression stability by stability values (M). The difference of the data between the two algorithms is the presented form. The first row of the table uses the gene name in geNorm and uses the sample name in NormFinder. As Figure 2 demonstrates, the M values of nine candidate reference genes with various treatments are ranked from high to low and directly presented in the figures. A lower M value represents better stability, and a higher M value denotes worse stability. Generally, a candidate gene with an M value under 1.5 can be regarded as a compatible reference gene. Since the PP2A had the lowest M value in all the five treatments, it should be considered the best reference gene according to geNorm. Furthermore, *TIP41* ranks in the top three in five of six treatments for its stability, with M values below 1.5. This result is consistent with the total group, which indicated that *TIP41* can be considered a suitable reference gene, as well as *PP2A.* Although *NCBP2* barely reaches the top in terms of individual treatments, it shows extraordinary stability among the group. This result is similar to that of the BestKeeper and NormFinder analysis. In addition, the geNorm also gives pairwise variation (Figure 3 and Table S3), aiming to optimize the number of required reference genes. The results are represented as the V-value. A V-value above the established threshold of 0.15 indicates that extra reference genes are necessary for data normalization [29]. Given that V-values are all above 0.15, an additional reference gene was necessary.

### 3.3. Comprehensive Analysis

**Figure 4:**
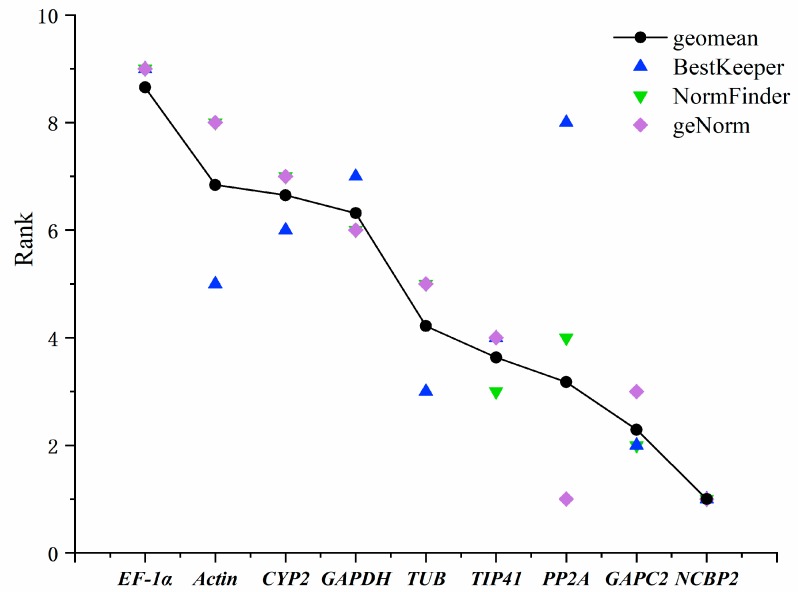
Comprehensive ranks of BestKeeper, NormFinder, and geNorm. The ranks by BestKeeper, NormFinder, and geNorm are exhibited in one graph. Geometric mean is also shown on the graph.

**Figure 5:**
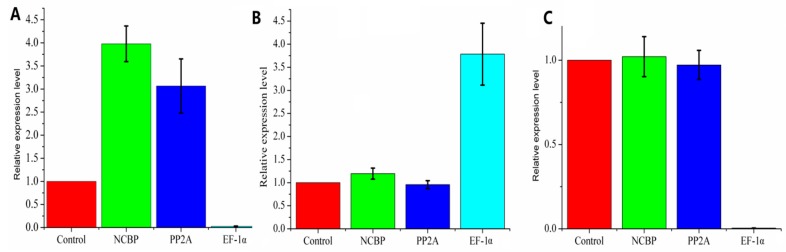
Validation of the reference genes. Relative expression level of 1-aminocyclopropane-1-carboxylate oxidase (ACO) was normalized using NCBP, protein phosphatase 2A (PP2A), and elongation factor 1-alpha (EF-1α), under different treatments. (**A**) Treated with NaCl. (**B**) Treated with MeJA. (**C**) Treated with CuSO_4_. Data are displayed as mean ± SEM (*n* = 3).

To validate the outcomes of the geNorm, NormFinder, and BestKeeper software, a comprehensive ranking platform RefFinder (https://www.heartcure.com.au/for-researchers/) was used to perform further validation. The final rankings are depicted in Table S4. A geometric mean was calculated based on the ranks of reference genes, and the results of the three methods floated around the geometric mean. In Figure 4, the results of geNorm, NormFinder, BestKeeper analysis and the geometric mean are depicted on a graph. Based on their comprehensive rank, NormFinder and geNorm tend to have better consistency compared to BestKeeper. *EF-1α*, *Actin*, *CYP2*, and *GAPDH* were not considered to be good reference genes according to the three methods (Table S5). Since the evaluating methods are different, this discrepancy is rational and acceptable. Furthermore, geNorm combined two stable reference genes for the best combination, though this combination should not be considered as two individual stable reference genes [29]. Overall, according to the validation, there is no doubt that *NCBP2* and *PP2A* can be selected as the best reference genes for *P. somniferum* under the tested conditions. In addition, the expression pattern of a target gene, 1-aminocyclopropane-1-carboxylate oxidase (*ACO*) was analyzed using the most stable and least stable reference genes according to our previous reports [13]. As depicted in Figure 5, the expression of *ACO* was steady in different treatments with *NCBP2* and *PP2A* as reference. While, when it normalized with *EF-1α*, the expression pattern was obviously overestimated. For instance, when it normalized with *EF-1α*, the expression level of *ACO* seemed to sharply decline in CuSO_4_ treatment, in fact, it was immune to CuSO_4_. Hence, an appropriate reference gene is critical for the normalization of target gene expression in plants.

## 4. Discussion

*P. somniferum* is attractive to researchers because of its ability to produce opiate compounds. Since the plant’s analgesic activity has already been confirmed, many studies have focused on exploring and understanding the biosynthetic pathway involved in alkaloid production [2,33,34]. Moreover, gene expression level plays an inevitable and significant role in studies on the metabolic pathway. Considering its high performance and availability, qPCR is frequently used in the high-throughput analysis of gene transcript level study. To ensure the accuracy and reliability of the data, a suitable reference gene is required. However, to our knowledge, there are no validated reference genes in *P. somniferum.* Therefore, in this study, the expression level stability of nine candidate reference genes for *P. somniferum* was thoroughly analyzed. Three frequently used Excel-based tools (BestKeeper, NormFinder, and geNorm) were employed for reference gene selection. Furthermore, in order to systematically investigate the stability of the candidate genes, six treatments, including osmotic press (PEG), mental stress (CuSO_4_), cold stress (4 °C), salt stress (NaCl), hormone treatment (MeJA), and one group without treatment (WT) as control were conducted [13,14]. The results showed that the expression of candidate reference genes was not stable in all the tested conditions and thus it is necessary to select different genes to normalize expression under different experimental conditions.

As an important factor, the expression level of the reference genes must be distributed in an advisable range and also have low Cp values. Herein, the expression level of nine candidate genes was determined, and the result was demonstrated as Cp values were listed in Figure 1. The mean Cp values are clearly distributed in a reliable range from 17.60 to 25.87, showing that the selected candidate genes have the potential to provide an accurate normalization [35]. Since a narrow range of distribution indicates lower variability and higher stability, *NCBP2* should be considered the best reference gene, with its Cp value distributed from 20.88 to 32.68. Additionally, the stability was confirmed by NormFinder and geNorm (Figure 3 and Table 2).

To further assess the stability of the candidate genes, three Excel-based mathematical tools were applied to demonstrate the data directly, according to the previous studies [36,37]. Given that each of the three chosen methods have unique methods of data processing, the discrepancy in outcomes is rational. Therefore, we combined the results of the three methods using Reffinder to determine the most stable reference genes and improve the credibility of our selection and validation. Meanwhile, since the gene expression stability varied under different conditions, four abiotic stresses, including drought stress, salt stress, cold stress, heavy metal stress, and hormone stress were selected based on previous studies [38,39].

According to the results of BestKeeper, *NCBP2* and *GAPC2* are the most stable reference genes in the entire group. These results exhibit no difference in the outcomes of NormFinder and geNorm for the top-ranked *NCBP2* but are discordant with the results acquired by geNorm. Considering the BestKeeper using the CV ± SD as the foundation of stability ranking, which is different from geNorm’s algorithm, this discrepancy is acceptable from a comprehensive aspect [36,40]. Despite the aforementioned differences, results of the three methods show great consistency in selecting the best reference gene. There was great consistency in the rankings of the results, demonstrating that *NCBP2* has the best stability (Figure 3). Additionally, a pairwise variation was conducted by geNorm. Since the V-values are above the suggested threshold of 0.15, the extra reference gene must be selected. Eventually, an online comprehensive analysis was also conducted, demonstrating that *NCBP2*, *GAPC2*, and *TIP41* possess the best rank. Therefore, we recommend the top three candidate genes- *NCBP2*, *GAPC2*, and *TIP41* as reference genes. Interestingly, although a commonly used reference gene, expression of *GAPDH* in *P. somniferum* was not stable enough for accurate normalization, but was recommended as one of the best reference genes in Barber [41], *E. konishii* Hayata [42], and sugarcane under a water deficit environment [27]. This difference demonstrates that no reference gene is universally available, so the selection and validation of suitable reference genes remain indispensable. Indeed, the expression stability of one species could vary under different environments. That is why we set five treatments in this study, and the results proved the necessity of this configuration. *TUB* was the most stable reference gene in the MeJA treatment. However, *TIP41* was most stable under salt stress, and *PP2A* expressed most stably under low temperature and drought situations. The same phenomenon is quite common and observed in many more publications than those reported in *Neolamarckia cadamba* [43], *Cynodon dactylon* [44], and *Agrostis stolonifera* [45], which further suggests that experimental conditions must be considered when analyzing gene expression.

## 5. Conclusions

In this study, nine candidate reference genes of *P. somniferum* were selected and their expression stabilities were investigated. The most stably expressed genes were able to normalize the data of RT-qPCR, which is a commonly used tool for gene expression analysis. According to the results of BestKeeper, NormFinder, and geNorm, *NCBP2*, *GAPC2*, *TIP41*, and *PP2A* were considered the most stable reference genes under all treatments, while *EF-1*, *Actin*, and *CYP2* have the least stability. The optimal number of required reference genes for normalization was also calculated by pairwise variation. The results reveal that more than one reference gene is necessary for accurate normalization. Overall, we suggest a combination of *NCBP2* and *PP2A* as the best reference genes for accurate normalization. To our best knowledge, this study is the first investigation of the reference genes in *P. somniferum* and provides a foundation for further molecular research on this plant.

## Figures and Tables

**Table 1:** Information of candidate reference genes and primers for RT-qPCR in *Papaver somniferum*.

Gene	Gene Name	PCR	Primer Sequences (5′-3′) (Forward/Reverse)	Length (bp)/Tm (°C)	PCR Efficiency
*Actin*	Actin-11	AT3G12110	GCTGTCCTTTCCCTCTACGC/AGGGCATCAGTAAGGTCACG	155/88.1	1.867
*CYP2*	Cyclophilin 2	AT4G33060	TGTGCTAATGCTGGAACGCC/TCTCGACCTCCGCCAAATTC	148/83.6	1.851
*EF-1α*	Elongation factor 1-α	AT5G60390	CCTGGACAGATAGGTAATGG/TAATACCAGCATCACCGTTC	135/86.8	1.885
*GAPC2*	Glyceraldehyde-3-phosphate dehydrogenase C2	AT3G04120	CCACAAATTGCCTTGCTCCC/TGGGTGGCAGTGATGGAGTG	97/82.7	1.871
*GAPDH*	Glyceraldehyde 3-phosphate dehydrogenase	AT1G42970	GCCAAGGCTGTGTCATTAGT/AAAGTCCCTTCTTCTCGACG	115/85.4	1.866
*NCBP2*	Nuclear cap binding protein 2	AT5G44200	AAACAGCAAAACCCCCTGTG/CCCCAATCAAAATCCACACG	129/84.2	1.885
*PP2A*	Protein phosphatase 2A gene	AT3G25800	TCTTCGTGCGGGTTGTTTCC/CGGATCTCCTGACCATTGGC	118/84.9	1.880
*TIP41*	TIP41-like protein	AT4G34270	GTTCCTGCTGCTGCCAAATG/GATGTTCCCTTCCCCCTCTG	135/82.2	1.884
*TUB*	Beta-6-tubulin	AT5G12250	TTGCTTCAGGACCCTCAAGC/GTTGCCCAGGGAACCTAAGG	107/85.8	1.887

**Table 2:** Expression stability rank of nine candidate genes by BestKeeper software (coefficient of variance (CV) ± standard deviation (SD)).

Rank	Cold	PEG	NaCl	MeJA	CuSO_4_	WT	Total
**1**	*GAPC2*	*NCBP2*	*NCBP2*	*TUB*	*Actin*	*NCBP2*	*NCBP2*
	5.93 ± 1.35	7.66±1.88	6.13 ± 1.45	6.96 ± 1.89	6.75 ± 1.76	9.24 ± 2.36	8.26 ± 2.04
**2**	*TIP41*	*CYP2*	*TUB*	*NCBP2*	*GAPDH*	*GAPC2*	*GAPC2*
	7.59 ± 1.88	10.58 ± 2.67	6.64 ± 1.65	7.51 ± 1.94	8.26 ± 1.90	9.92 ± 2.21	8.99 ± 2.00
**3**	*Actin*	*TUB*	*TIP41*	*GAPC2*	*TUB*	*CYP2*	*TUB*
	8.48 ± 2.04	11.08 ± 2.91	7.44 ± 1.75	7.95 ± 1.84	8.27 ± 2.14	10.15 ± 2.75	9.05 ± 2.37
**4**	*NCBP2*	*TIP41*	*GAPC2*	*TIP41*	*GAPC2*	*TUB*	*TIP41*
	8.70 ± 2.17	11.46 ± 2.85	7.57 ± 1.56	8.10 ± 2.07	8.77 ± 1.94	11.16 ± 2.88	9.49 ± 2.34
**5**	*TUB*	*PP2A*	*CYP2*	*PP2A*	*NCBP2*	*GAPDH*	*ACTIN*
	9.14 ± 2.45	11.65 ± 2.67	8.92 ± 2.21	9.39 ± 2.37	10.07 ± 2.46	11.59 ± 2.49	10.75 ± 2.69
**6**	*PP2A*	*Actin*	*GAPDH*	*GAPDH*	*TIP41*	*PP2A*	*CYP2*
	9.31 ± 2.18	12.35 ± 3.20	9.35 ± 1.91	11.48 ± 2.60	10.34 ± 2.49	11.92 ± 2.85	10.78 ± 2.76
**7**	*CYP2*	*GAPC2*	*PP2A*	*Actin*	*CYP2*	*Actin*	*GAPDH*
	9.72 ± 2.54	12.73 ± 2.85	9.72 ± 2.18	11.51 ± 2.83	11.20 ± 2.85	12.93 ± 3.22	10.97 ± 2.42
**8**	*GAPDH*	*GAPDH*	*Actin*	*CYP2*	*PP2A*	*TIP41*	*PP2A*
	11.27 ± 2.45	12.93 ± 2.95	12.84 ± 3.18	12.36 ± 3.21	11.48 ± 2.65	12.97 ± 3.51	11.13 ± 2.61
**9**	*EF-1*	*EF-1*	*EF-1*	*EF-1*	*EF-1*	*EF-1*	*EF-1*
	26.25 ± 4.55	29.27 ± 4.98	25.23 ± 4.04	30.66 ± 5.83	23.21 ± 4.23	29.51 ± 4.71	27.64 ± 4.81

**Table 3:** Expression stability rank of nine candidate genes by NormFinder software.

Rank	NaCl	PEG	Cold	MeJA	CuSO_4_	WT	Total
**1**	*TIP41*	*PP2A*	*PP2A*	*TUB*	*GAPC2*	*CYP2*	*NCBP2*
	0.228	0.136	0.627	0.339	0.189	0.569	0.608
**2**	*NCBP2*	*CYP2*	*TIP41*	*TIP41*	*NCBP2*	*GAPC2*	*GAPC2*
	0.228	0.222	0.651	0.364	0.200	0.690	0.615
**3**	*GAPC2*	*TIP41*	*CYP2*	*NCBP2*	*TUB*	*GAPDH*	*TIP41*
	0.394	0.389	0.677	0.504	0.339	0.873	0.643
**4**	*TUB*	*GAPDH*	*GAPC2*	*GAPC2*	*TIP41*	*TIP41*	*PP2A*
	0.450	0.589	0.698	0.664	0.437	1.214	0.750
**5**	*CYP2*	*NCBP2*	*TUB*	*PP2A*	*PP2A*	*NCBP2*	*TUB*
	0.588	0.590	0.892	0.769	0.488	1.282	1.028
**6**	*GAPDH*	*GAPC2*	*NCBP2*	*GAPDH*	*CYP2*	*PP2A*	*GAPDH*
	0.654	0.819	1.032	1.449	0.628	1.482	1.136
**7**	*PP2A*	*TUB*	*Actin*	*Actin*	*GAPDH*	*Actin*	*CYP2*
	0.685	0.919	1.422	1.695	0.890	1.657	1.410
**8**	*Actin*	*Actin*	*GAPDH*	*CYP2*	*Actin*	*TUB*	*Actin*
	2.465	3.211	1.517	3.008	2.177	3.565	2.317
**9**	*EF-1*	*EF-1*	*EF-1*	*EF-1*	*EF-1*	*EF-1*	*EF-1*
	3.731	3.286	3.523	4.774	3.142	4.155	3.694

## Supplementary Materials

The following are available online at https://www.mdpi.com/2073-4425/11/2/124/s1. Table S1: The sequences used for primer design. Table S2: The PCR products sequencing data of nine reference genes. Table S3: Pairwise variation (Vn/n+1) analysis of nine candidate reference genes calculated by geNorm. Table S4: Expression stability values of nine candidate genes calculated by RefFinder in *P. somniferum*. Table S5: Rankings of nine candidate reference genes based on stability values calculated by four kinds of software. Figure S1: The agarose gel electrophoresis of PCR products using nine reference genes. Figure S2: Melting curves of the nine candidate reference genes.
