# Supplementary material for: Selection of the Reference Gene for Expression Normalization in Papaver somniferum L. under Abiotic Stress and Hormone Treatment
Source: Genes (Basel). 2020 Jan 23;11(2):124. doi: 10.3390/genes11020124 (PMC7074096; doi:10.3390/genes11020124)
Supplement: Supplementary file 1 [file genes-11-00124-s001.zip › Supplemental File.doc]

**Supplementary Materials: Selection of the Reference Gene for Expression Normalization in** ***Papaver Somniferum* under Abiotic Stress and Hormone Treatment**

Zhaoping Zhang ^1^, Changjian Li ^2^, Junqing Zhang ^2^, Fang Chen ^1^, Yongfu Gong ^1^, Yanrong Li ^1^, Yujie Su ^1^, Yujie Wei ^1^ and Yucheng Zhao ^2,^*

**Supplementary Figures**

**Figure S1.** The agarose gel electrophoresis of PCR products using nine reference genes. 1-9 represent *actin, GAPDH, CYP2, EF-1α, GAPC2, NCBP2, PP2A, TIP41*, and *TUB*, respectively.

**Figure S2.** Melting curves of the nine candidate reference genes.

**Table S1.** The sequences used for primer design and their PCR products sequencing data. 511-431 represents different tissue and growth cycles.

**See supporting excel file.**

**Table S2.** The PCR products sequencing data of nine reference genes.

1. *Actin*：

GCTGTCCTTTCCCTCTACGCCAGTGGTCGAACAACTGGTATTGTTCTCGACTCTGGTGATGGTGTCAGCCACACAGTACCCATCTACGAAGGTTATGCACTCCCACATGCCATCTTCTCTACGTACGGGCTGTCCTTTCCCTCTACGCCAGTGGTCGAACAACTGGTATTGTTCTCGACTCTGGTGATGGTGTCAGCCACACAGTACCCATCTACGAAGGTTATGCACTCCCACATGCCATCTTCTCTACGTACGG

2. *CYP2*

TTTGCTTCAGGACCCTCAAGCTAACACACTCCTAGCTTCTGGTGATCTGAACCATCTTATCTCTGCTATGCATGAGGCTTCGATTTGCTTCAGGACCCTCAAGCTAACACACTCCTAGCTTCTGGTGATCTGAACCATCTTATCTCTGCTATGCATGAGGCTTCGA

3. *EF-1α:*

TTTGCTTCAGGACCCTCAAGCTAACACACTCCTAGCTTCTGGTGATCTGAACCATCTTATCTCTGCTATGCATGAGGCTTCGATTTGCTTCAGGACCCTCAAGCTAACACACTCCTAGCTTCTGGTGATCTGAACCATCTTATCTCTGCTATGCATGAGGCTTCGA

4. *GAPC2*:

CATAAGCCACCATGAAGATTTGGATTATTGAAGGCCTTATGACTACCGTTCACTCCATCACTGCCACCCAA

5. *GAPDH*

GCCAAGGCTGTGTCATTAGTCTTACCTCAATTGAAGGGAAAGCTTAACGGCATTGCACTTAGAGTTCCTACTCCTAATGTTTCTGTAGTTGATCTAGTTATTAACGTCGAGAAGAAGGGACTTTA

6. *NCBP2*

TAAACAGCAAAACCCCCTGTGGCTTTTGCTTTATCTATGTACTACTCCAGAGATGTAAACGAGCGATGCAGTCAAGATAAGTGGGACAATTCTGGACGATCGCCCAATTCGTGTGGATTTTGATTGGGG

7. *PP2A*

TTCTTCGTGCGGGTTGTTTCCCATTGCTTACCCTAGTGCTCCAGAAATGTTGAAGACAGACAGAACTCAGGGTCTATCTACAGCCAGTTGTGTCAAGATGACATGCCAATGGTCAGGAGATCCG

8. *TIP41*

TGTTCCTGCTGCTGCCAAATGGAAATTCAGAAGCAAACTTTTTCAGCAGGTCATATTTGATTATGACTATACCTTACTACTCCATACTTTGGAAAGTGAAACAGTTGAAAAGAATACAGAGGGGGAAGGGAACATCA

9. *TUB*

TTTGCTTCAGGACCCTCAAGCTAACACACTCCTAGCTTCTGGTGATCTGAACCATCTTATCTCTGCTACCATGAGTGGCGTTACCTGCTGCCTTAGGTTCCCTGGGCAACA

**Table S3. Pairwise variation (Vn/n+1) analysis of nine candidate reference genes calculated by geNorm software.**

| Treatments | Pairwise variation (V) values | | | | | | |
| --- | --- | --- | --- | --- | --- | --- | --- |
|  | V2/3 | V3/4 | V4/5 | V5/6 | V6/7 | V7/8 | V8/9 |
| NaCl | 0.346 | 0.234 | 0.169 | 0.162 | 0.151 | 0.418 | 0.601 |
| PEG | 0.215 | 0.233 | 0.214 | 0.214 | 0.182 | 0.573 | 0.532 |
| Cold | 0.348 | 0.348 | 0.298 | 0.272 | 0.263 | 0.301 | 0.568 |
| MeJA | 0.351 | 0.365 | 0.289 | 0.315 | 0.374 | 0.511 | 0.769 |
| CuSO4 | 0.278 | 0.172 | 0.177 | 0.155 | 0.189 | 0.375 | 0.506 |
| WT | 0.223 | 0.334 | 0.64 | 0.441 | 0.329 | 0.603 | 0.673 |

**Table S4.** Expression stability values of 9 candidate genes calculated by the RefFinder in *P. Somniferum*

| **Rank** | **Control** | **Cold** | **MeJA** | **PEG** | **NaCl** | CuSO4 | **Total** |
| --- | --- | --- | --- | --- | --- | --- | --- |
| **1** | GAPC2 | PP2A | TUB | PP2A | NCBP2 | NCBP2 | GAPC2 |
|  | 1.41 | 1.5 | 1.57 | 1.32 | 1.68 | 2.51 | 1.57 |
| **2** | CYP2 | CYP2 | NCBP2 | TIP41 | TIP41 | GAPC2 | NCBP2 |
|  | 1.86 | 2.63 | 2.06 | 2.21 | 2.21 | 2.71 | 1.57 |
| **3** | GAPDH | GAPC2 | GAPC2 | CYP2 | GAPC2 | TIP41 | TIP41 |
|  | 2.28 | 2.63 | 2.78 | 2.78 | 3.22 | 3.46 | 2.91 |
| **4** | NCBP2 | TIP41 | PP2A | NCBP2 | CYP2 | PP2A | PP2A |
|  | 3.98 | 2.99 | 3.34 | 3.64 | 3.64 | 3.64 | 3.13 |
| **5** | TIP41 | TUB | TIP41 | GAPDH | TUB | TUB | TUB |
|  | 4.76 | 4.79 | 3.36 | 4.28 | 3.94 | 3.94 | 4.73 |
| **6** | PP2A | NCBP2 | ACT | TUB | PP2A | CYP2 | GAPDH |
|  | 5.73 | 5.42 | 6.48 | 5.63 | 3.98 | 4.12 | 5.73 |
| **7** | ACT | ACT | GAPDH | GAPC2 | GAPDH | ACT | CYP2 |
|  | 7 | 6.05 | 6.48 | 5.96 | 6.19 | 4.76 | 7.24 |
| **8** | TUB | GAPDH | CYP2 | ACT | ACT | GAPDH | ACT |
|  | 7.44 | 6.96 | 8 | 8 | 8 | 5.12 | 7.74 |
| **9** | EF-1α | EF-1α | EF-1α | EF-1α | EF-1α | EF-1α | EF-1α |
|  | 9 | 9 | 9 | 9 | 9 | 9 | 9 |

**Table S5. Rankings of nine candidate reference genes based on stability values calculated by four softwares.**

| Rank | | 1 | 2 | 3 | 4 | 5 | 6 | 7 | 8 | 9 |
| --- | --- | --- | --- | --- | --- | --- | --- | --- | --- | --- |
| **Control** | geNorm | PP2A/NCBP2 | GAPC2 | TIP41 | TUB | GAPDH | CYP2 | ACT | EF-1α | / |
|  | NormFinder | CYP2 | GAPC2 | GAPDH | TIP41 | NCBP2 | PP2A | ACT | TUB | EF-1α |
|  | BestKeeper | NCBP2 | GAPC2 | CYP2 | TUB | GAPDH | PP2A | ACT | TIP41 | EF-1α |
|  | RefFinder | GAPC2 | CYP2 | GAPDH | NCBP2 | TIP41 | PP2A | ACT | TUB | EF-1α |
| **Cold** | geNorm | CYP2/PP2A | TUB | GAPC2 | TIP41 | NCBP2 | GAPDH | ACT | EF-1α | / |
|  | NormFinder | PP2A | TIP41 | CYP2 | GAPC2 | TUB | NCBP2 | ACT | GAPDH | EF-1α |
|  | BestKeeper | GAPC2 | TIP41 | ACT | NCBP2 | TUB | PP2A | CYP2 | GAPDH | EF-1α |
|  | RefFinder | PP2A | CYP2 | GAPC2 | TIP41 | TUB | NCBP2 | ACT | GAPDH | EF-1α |
| **MeJA** | geNorm | NCBP2/PP2A | TUB | TIP41 | GAPC2 | ACT | GAPDH | CYP2 | EF-1α | / |
|  | NormFinder | TUB | TIP41 | NCBP2 | GAPC2 | PP2A | GAPDH | ACT | CYP2 | EF-1α |
|  | BestKeeper | TUB | NCBP2 | GAPC2 | TIP41 | PP2A | GAPDH | ACT | CYP2 | EF-1α |
|  | RefFinder | TUB | NCBP2 | GAPC2 | PP2A | TIP41 | ACT | GAPDH | CYP2 | EF-1α |
| **PEG** | geNorm | PP2A/TIP41 | GAPDH | TUB | CYP2 | GAPC2 | NCBP2 | ACT | EF-1α | / |
|  | NormFinder | PP2A | CYP2 | TIP41 | GAPDH | NCBP2 | GAPC2 | TUB | ACT | EF-1α |
|  | BestKeeper | NCBP2 | CYP2 | TUB | TIP41 | PP2A | ACT | GAPC2 | GAPDH | EF-1α |
|  | RefFinder | PP2A | TIP41 | CYP2 | NCBP2 | GAPDH | TUB | GAPC2 | ACT | EF-1α |
| **NaCl** | geNorm | CYP2/PP2A | TIP41 | NCBP2 | TUB | GAPC2 | GAPDH | ACT | EF-1α | / |
|  | NormFinder | TIP41 | NCBP2 | GAPC2 | TUB | CYP2 | GAPDH | PP2A | ACT | EF-1α |
|  | BestKeeper | NCBP2 | TUB | TIP41 | GAPC2 | CYP2 | GAPDH | PP2A | ACT | EF-1α |
|  | RefFinder | NCBP2 | TIP41 | GAPC2 | CYP2 | TUB | PP2A | GAPDH | ACT | EF-1α |
| CuSO**_4_** | geNorm | CYP2/PP2A | TIP41 | NCBP2 | TUB | GAPC2 | GAPDH | ACT | EF-1α | / |
|  | NormFinder | GAPC2 | NCBP2 | TUB | TIP41 | PP2A | CYP2 | GAPDH | ACT | EF-1α |
|  | BestKeeper | ACT | GAPDH | TUB | GAPC2 | NCBP2 | TIP41 | CYP2 | PP2A | EF-1α |
|  | RefFinder | NCBP2 | GAPC2 | TIP41 | PP2A | TUB | CYP2 | ACT | GAPDH | EF-1α |
